# Development of the Teeth

**Published:** 1888-08

**Authors:** R. Nicol


### Development of the Teeth.

BY R. NICOL.

All teeth are developed from a part of the mucous membrane, and any connection they may afterwards acquire with the bone as their retention by gomphosis in the maxillary bones is a secondary matter. Teeth then may correctly be regarded as "dermal appendages" or even "calcified hairs." In order, however, to understand how they may be developed from this membrane it is necessary to have a perfect knowledge of its histology. All of the mucous membranes have cavities or tubes communicating with the exterior by the different openings in the body and are indeed a part of the external skin with which they blend, as at the lips, etc.; but these membranes in different situations present important peculiarities in structure. There are two distinct classes of mucous membrane, the one being provided with pavement epithelium and the other with columnar.

There is quite a difference of opinion among writers with regard to the number of layers into which the mucous membrane may be divided, but the following diagram includes all the layers with the various names given to each of them by different histologists. The diagram is not drawn to any scale so the thickness of the different layers is not to be inferred from the space assigned to each.

DIAGRAM OF THE MUCOUS MEMBRANES.

Epidermis. Epithelium.

Corneous layer, (composed of dead epithelial cells.)
Malpighian layer (columnar, cylindrical or prismatic cells) constitutes peripheral portion of enamel organ.

Basement Membrane, Intermediate Membrane, or Membrana Praeformativa (separates dentine bulb in enamel organ.)

Dermis Proper. Mucous Membrane. Mucosa. Mucous Layer. Cutis Vera. Chorion or Corium.

Pars Papillaris or papillary part.
From this arises the dentine bulb.
Pars recticularis or nether part.
Subdermis or submucous layer.
Some say the dentine bulb arises from this layer.

There is a great deal of uncertainty about the basement membrane. Some writers, Tomes for one, denies its existence while those who do believe in it, dispute about its location.

The chorion is made up of inelastic and elastic fibrous tissues, a few fibro-plastic elements, with capillaries, lymphatics, and nerves. The epidermis or epithelium is made up of two layers the corneous and the Malpighian and one of its characteristic properties is, that it is regenerated by the formation of new cells from below, the effete structures or dead cells forming the corneous layer being thrown off. Mucous membrane is provided with follicular glands extending through its entire thickness and terminating in rounded extremities upon the submucous tissue.

Such is the tissue from which the teeth are developed. Before the fortieth or forty-fifth day of intra-uterine life there is no sign of teeth in the human jaw, but about this time there appears a heaping up of the epithelial cells all along the whole jaw. This is called the epithelial ridge. Beneath this external ridge a projection sinks into the subjacent elements, the outline or form of which nearly represents the form of the letter V, with the apex slightly towards the inner or linguial side. This is the epithelial band or groove, and it is lined with the latest formed or bottom layer of cells viz.: the cylindrical columnar, or prismatic cells of the Malpighian layer. This band or groove is convex on its external or buccal surface, and concave on its inner surface.

Half way down the groove another inbending or offshoot is given off from its inner surface which is called the epithelial lamina and is lined with the same sort of cells as the groove. This offshoot or lamina is a continuous process extending the whole length of the epithelial band.

The enamel organ, the first trace of the dental follicle, originates from points upon the free extremity of this lamina. They show themselves as slight tubercles or buds at intervals along the margin of the lamina corresponding in number and situation to the temporary teeth. These tubercles are called the primitive. In continuing its evolution the first bud which was at first spherical, becomes cylindrical and lengthens out into the first cord. The cord after changing its course from a horizontal to a vertical direction becomes larger at its extremity club-shaped in form and acquires greater size. This results from the multiplication of the polyhedral cells of which it is composed, and from the increase in the number of prismatic cells which form its outer layer. This body assumes a somewhat spherical form and represents the first enamel organ or the enamel organ of the temporary tooth.

At this time the dentine bulb makes it appearance being developed from the papillary layer of the chorion. It grows or pushes up into the enamel organ causing it to assume the form of a hood. The dental bulb first appears as a rounded nipple of hemispheric form, its convexity corresponding with the concavity of the enamel organ. This little papilla or incipient dental bulb is composed at first of nucleated embryo-plastic elements, and afterwards of fusiform and stellate bodies. At this time a vascular loop enters its substance and the bulb now shows a tendency to take the form of the future tooth. The dentine bulb then thus determines the contour or form of the crown of the future tooth. This is the theory held by many, but Magitot asserts that the enamel organ is the main factor in determining the location and form of the tooth. All teeth whether covered with enamel or not are in their formative stage endowed with an enamel organ.

Such is the origin of the follicles of the twenty temporary teeth. All of the twenty permanent teeth which succeed the twenty temporary teeth are developed in the same manner. At the point where the first or primitive cord merges into the enamel organ a bourgeon or bud will be found from which is developed the enamel organ of the permanent tooth. Legros, Magitot, Tomes, Kolliker, Waldeyer, Frey, Owen, Wedl, and others assert that this bud originates as above described in the cord, while others, among whom are Dr. J. E. Ondit, a French writer, and Dr. Williams, of New Haven, hold that the follicles of the twenty teeth which succeed the temporary teeth emanates directly and independently from the mucous membrane.

The elements of which this bud is composed resembles those of the primitive bud viz.: polygonal cells in the centre covered with a layer of prismatic cells. This bud sinks to the bottom of the dental groove where it soon loses its connection with the primitive follicle though still retaining its connection with the lamina. The descent of the secondary bud into the groove is soon followed by the development of a secondary cord and enamel organ. This cord passes downward between the oseous wall and the primitive follicle on the internal or lingual surface of the latter. The follicles of the permanent teeth become isolated losing all connection with the epithelial lamina from the period at which the bulb begins to assume the forms of the respective teeth. After this rupture the cord lengthens and buds out into a plexus, but finally atrophies and disappears. Its failure to do this, is, by some, considered to be the cause of supernumerary teeth.

The follicle of the first permanent molar is derived from an epithelial cord which originates directly from the epithelial lamina. The follicle of the second molar is a bud from the cord of the first molar, and the follicle of the third molar is derived from the cord of the second.

